# Simulation-to-Real Trip-Fall Detection with Continuous-Wave Doppler Radar via Physics-Informed Kinematic Modeling and Domain Randomization

**DOI:** 10.3390/s26103211

**Published:** 2026-05-19

**Authors:** Kosuke Okusa

**Affiliations:** Department of Data Science for Business Innovation, Chuo University, Tokyo 112-8551, Japan; okusa@kc.chuo-u.ac.jp; Tel.: +81-3-3817-1927

**Keywords:** continuous-wave (CW) doppler radar, domain randomization, physics-informed simulation, privacy-preserving sensing, simulation-only training, simulation-to-real transfer, time–frequency analysis, trip-fall detection

## Abstract

Falls among older adults are a major public health concern, yet collecting large-scale real fall data for radar-based detection is ethically and practically difficult. This study presents a controlled simulation-to-real feasibility study for trip-fall detection using continuous-wave (CW) Doppler radar. The method couples a physics-informed kinematic trip-fall model with a CW radar observation model to synthesize I/Q signals and Doppler spectrograms, while domain randomization varies body size, fall direction, initial velocity, sensor placement, aspect angle, amplitude, and noise. Synthetic walking and respiration data were also generated for controlled three-class classification among trip fall, walking, and seated quiet breathing. In Experiment I, the simulated spectrograms reproduced the dominant time–frequency characteristics of measured enacted trip-fall signals acquired with a 24 GHz CW radar; quantitative similarity analysis yielded a mean SSIM of 0.782 and a Doppler-ridge MAE of 24.6 Hz across five fall directions. In Experiment II, a ResNet-18 classifier trained only on simulated spectrograms achieved a macro-F1 score of 0.912 [95% CI: 0.883–0.936] on measured data from ten participants, three start locations, and eight directions. Under the present controlled evaluation, this exceeded the available real-data-trained baseline of 0.748 [95% CI: 0.691–0.805] (paired subject-level permutation test, p=0.006). These findings suggest that physics-informed simulation with domain randomization can reduce dependence on real trip-fall samples under limited-data conditions. The results do not establish robustness to other fall morphologies, fall-like activities of daily living, different environments, different radar devices, or embedded deployment.

## 1. Introduction

With the rapid progression of global population aging, falls have become a significant public-health concern for community-dwelling older adults, leading to mortality, serious injury, hospitalization, transition to long-term care, and diminished quality of life (QOL). In the United States, annual medical expenditures attributable to falls among older adults are estimated to reach tens of billions of dollars [1]. From a global disease-burden perspective, falls remain a leading contributor to morbidity and mortality [2]. Accordingly, there is an urgent need for fall-detection technologies that ensure early detection, allow rapid intervention, and balance high reliability with public acceptability.

Existing approaches to fall detection can be broadly categorized into contact (wearable) and non-contact (environmentally installed) methods. On the wearable side, studies range from simple thresholding with inertial sensors (accelerometers and gyroscopes) to advanced machine and deep learning models [3,4,5,6]. However, practical constraints persist, including adherence during daily use, sensitivity to placement, limited battery life, and blind spots caused by removal or misplacement [3,6]. Among non-contact methods, floor-vibration systems classify falls using piezoelectric transducers [7], but their performance is highly dependent on floor structure and installation, often requiring site-specific calibration [8]. Vision-based approaches provide rich information and have benefited from CNNs, yet privacy concerns in sensitive spaces (e.g., bathrooms/bedrooms) and dependence on lighting conditions hinder adoption [9,10,11,12]. Furthermore, device-free approaches using Wi-Fi channel state information (CSI) demonstrate promise [13,14], but performance is highly influenced by network configuration and radio environment, necessitating retraining or additional calibration when deployed in new environments [14].

Against this background, microwave radar has garnered attention as a sensing modality that is privacy-preserving and independent of lighting and explicit subject consent (e.g., face exposure). The micro-Doppler effect—frequency modulations induced by micro-motions—encodes salient kinematic structure [15] and has been used for human activity recognition, tracking, gait analysis, and fall detection [16]. Furthermore, radar signatures are highly sensitive to aspect angle (the relative orientation between the radar’s line of sight and the subject’s motion vector) and sensor placement [16]; even moderate changes in aspect can materially alter time–frequency features and degrade classification accuracy [17]. Interference from non-target micro-motions (e.g., fans) and variations across indoor and outdoor environments can further impact performance [18,19].

Prior work on radar-based fall detection covers continuous-wave (CW) Doppler, FMCW, and IR-UWB systems. For CW radar, falls have been identified using Doppler time–frequency representations and wavelet-based analysis [20,21], yet accuracy is highly dependent on aspect and installation [17]. For FMCW radar, deep models applied to range–Doppler maps and point-cloud representations have enabled fall detection, fall direction estimation, and even multi-person tracking [22,23,24,25,26,27]; for instance, point-cloud enhancement can achieve high accuracy with new subjects and environments [24], although such methods usually assume environment-specific data collection and retraining. IR-UWB systems likewise achieve strong detection performance using CNNs or ConvLSTMs [28] but remain susceptible to installation, occlusion, and multipath, therefore requiring expanded training datasets to account for diverse geometries [23,28].

A fundamental barrier is that falls are inherently rare and hazardous, making large-scale acquisition of genuine fall data ethically and practically difficult. Consequently, many datasets rely on enacted (simulated) falls; however, domain shift between enacted and real-world falls raises concerns regarding external validity [4,29,30]. Public datasets (e.g., SisFall) are valuable resources [31] but may not fully cover variability in subject demographics, living environments, and fall morphologies (direction, posture transitions, assistive devices), and performance often deteriorates when applied to new environments or orientations [4,30]. Moreover, radar micro-/range–Doppler signatures depend on sensor placement × room layout × fall direction [16,17]; attempting to comprehensively cover this combinatorial space with measurements—and retraining models at each site—poses a significant practical challenge.

To address this bottleneck, we pursue a simulation-driven learning approach. We ground our method in the observation model of a CW microwave Doppler radar and generate radar observations—I/Q, phase/Doppler frequency, and Doppler spectrograms—from a physics-informed kinematic model of trip-fall motion. In radar recognition, micro-Doppler synthesis using motion capture or physics models, GAN-based waveform generation, and video-to-radar translation have shown promise for data diversification and sim-to-real transfer [32,33,34]. These approaches, however, typically require motion-capture data, real radar samples for generative training, or synchronized video observations. By contrast, the proposed framework generates CW Doppler signals from an analytic trip-fall kinematic model coupled with a radar observation model. The novelty is therefore not high-fidelity human animation or a new deep architecture, but a low-cost fall-specific simulation-to-learning pipeline that can produce training data without collecting real fall, motion-capture, or video data.

The present study addresses the following research questions. RQ1: Can a simplified physics-informed trip-fall model reproduce the dominant time–frequency characteristics of measured CW Doppler radar signals? RQ2: Can a classifier trained only on synthetic radar spectrograms detect measured trip falls under variations in subject position and fall direction? RQ3: How much do domain randomization and geometric parameter sweeps contribute to simulation-to-real transfer within the evaluated setting? RQ4: What limitations remain with respect to fall morphology, fall-like activities of daily living, environmental variability, radar-device variability, and deployment?

Our main contributions are as follows: (1) we propose a physics-informed simulator that couples a kinematic trip-fall model with a CW Doppler observation model; (2) we add quantitative simulation-to-real validation of measured and simulated spectrograms using SSIM, Doppler-ridge error, peak-Doppler error, and spectral-centroid error; (3) we show, under a controlled three-class task, that a model trained exclusively on simulated data can detect measured trip falls without using real fall data for training; (4) we report confidence intervals, subject-level statistical testing, fall-vs-nonfall metrics, and sensitivity/ablation results to clarify the contribution of the proposed fall model; and (5) we explicitly delimit the scope of the present results to enacted trip falls, one radar device, one measurement environment, and the activity classes considered here.

The remainder of this paper is structured as follows. Section 2 provides an overview of the operating principle and observation model of a CW Doppler radar. Section 3 describes the proposed fall detection model and simulation pipeline in detail. Section 4 explains the two experiments conducted in this study to validate the proposed method. Section 5 reports the results of Experiments I–II, including measured–simulated spectrogram comparisons, three-class discrimination among fall, walking, and seated quiet breathing under cross-subject and cross-location settings. Section 6 discusses the evaluation results, limitations of the proposed method, and future directions. Finally, Section 7 summarizes the study.

## 2. Overview of the Microwave Doppler Sensor

This section summarizes the operating principle of the Doppler sensor and its output signals. A Doppler sensor is a module that illuminates a target with electromagnetic waves and outputs a signal encoding the target’s motion. The sensor transmits a continuous wave toward the target; the frequency of the wave reflected from the target is shifted in proportion to the target’s radial velocity because of the Doppler effect. The sensor receives the echo and, via in-phase and quadrature (I/Q) detection against the transmitted reference, produces baseband I/Q signals which represent the phase of the transmit–receive pair.

A mathematical model of the sensor is provided below. Let $v_{s}\left(t\right)$ denote the transmitted signal with carrier frequency $f_{0}$ and initial phase $o_{s}$:

(1) $$\begin{array}{c} v_{s}\left(t\right)=A_{s}\cos\varphi_{s} \end{array}$$

where $\varphi_{s}=2\pi f_{0}t+o_{s}$, and $A_{s}$ is the transmit amplitude. The round-trip (RT) propagation time is

(2) $$\begin{array}{c} Tr\left(t\right)=\frac{2D(t)}{c}=\frac{2}{c}\left(l_{0}+\int_{0}^{t}v\left(t\right)\;dt\right), \end{array}$$

where *c* is the speed of light, D(t) is the instantaneous range between the sensor and target, $l_{0}$ is the initial range, and v(t) is the radial velocity. (We invoke the standard start–stop approximation and neglect range change within the RT interval.)

The received signal $v_{r}\left(t\right)$ is delayed by the RT time in (2) and can be expressed as

(3) $$\begin{array}{c} v_{r}\left(t\right)=A_{r}\cos\varphi_{r} \end{array}$$

with $\varphi_{r}=2\pi f_{0}\left(t-Tr(t)\right)+o_{s}$ and receive amplitude $A_{r}$. After I/Q demodulation, the sensor outputs

(4) $$\begin{array}{ccc} I(t) & = & \frac{A_{s}A_{r}}{2}\left(\sin\left(\varphi_{s}-\varphi_{r}\right)+\sin\left(\varphi_{s}+\varphi_{r}\right)\right)\\ Q(t) & = & \frac{A_{s}A_{r}}{2}\left(\cos\left(\varphi_{s}-\varphi_{r}\right)+\cos\left(\varphi_{s}+\varphi_{r}\right)\right) \end{array}$$

where $\varphi_{s}-\varphi_{r}$ represents the phase difference carrying low-frequency motion and range information, whereas $\varphi_{s}+\varphi_{r}$ resides near twice the carrier and is removed by low-pass filtering as it primarily contributes noise. Hence,

(5) $$\begin{array}{c} \begin{array}{c} I^{*}\left(t\right)=\frac{A_{s}A_{r}}{2}\sin\left(\varphi_{s}-\varphi_{r}\right),\\ Q^{*}\left(t\right)=\frac{A_{s}A_{r}}{2}\cos\left(\varphi_{s}-\varphi_{r}\right). \end{array} \end{array}$$

The instantaneous phase $\varphi_{d}$ and Doppler frequency $f_{d}$ of (5) satisfy

(6) $$\begin{array}{c} \begin{array}{cc} \varphi_{d} & =\varphi_{s}-\varphi_{r}=2\pi f_{0}Tr\left(t\right)=\frac{4\pi f_{0}}{c}D\left(t\right),\\ f_{d} & =\frac{1}{2\pi }\frac{d\varphi_{d}}{dt}=\frac{2f_{0}}{c}v\left(t\right). \end{array} \end{array}$$

Equation (6) depicts that the baseband phase is proportional to range D(t) and the instantaneous frequency is proportional to radial velocity v(t). Therefore, synthesizing sensor outputs reduces to modeling D(t).

The transmit/receive amplitudes depend on radiated power, antenna gain, target reflectivity, propagation loss, receiver gain, and measurement noise. These factors affect amplitude and SNR, whereas the Doppler phase in (6) is determined by the effective range evolution. We therefore synthesize the complex baseband signal as

(7) $$\begin{array}{c} \begin{array}{cc} I^{*}\left(t\right) & =\alpha \left(t\right)\sin\left\{\varphi_{d}\left(t\right)\right\}+n_{I}\left(t\right),\\ Q^{*}\left(t\right) & =\alpha \left(t\right)\cos\left\{\varphi_{d}\left(t\right)\right\}+n_{Q}\left(t\right), \end{array} \end{array}$$

where α(t) represents amplitude effects such as target reflectivity, antenna/receiver gain, and range-dependent attenuation, and $n_{I}\left(t\right)$ and $n_{Q}\left(t\right)$ denote additive noise. This separation is important because reflectivity changes the observed amplitude and SNR but does not directly scale the physical range that appears in the Doppler phase.

## 3. Mathematical Model for Fall Discrimination

This section highlights the mathematical model used for fall discrimination. As discussed in Section 2, simulating the Doppler sensor output requires a mathematical model for the target range D(t).

### 3.1. Fall Model

**Figure 1:**
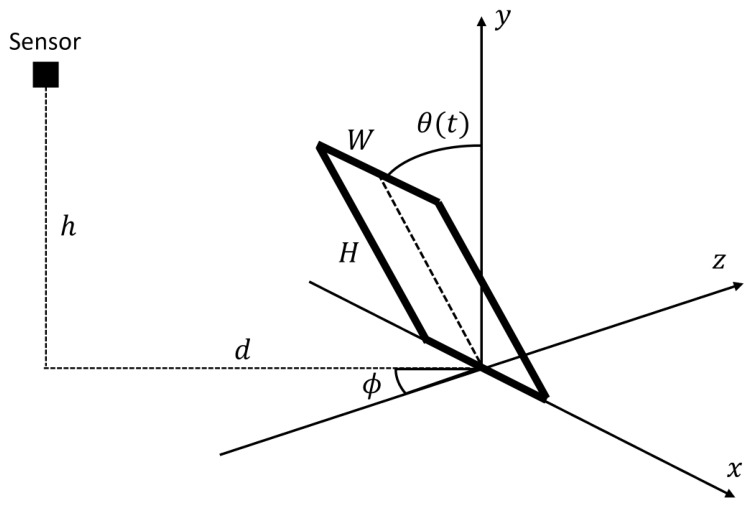
Schematic Representation of the Proposed Trip-Fall Model.

For clarity, we model the human body as a rectangular plate of width *W* and height *H* (Figure 1) and describe its fall kinematics.

The rectangular-plate approximation is intentionally minimalist. The aim is to reproduce the dominant radial-velocity evolution and the corresponding Doppler ridge generated by rapid body descent, not to provide a full biomechanical or electromagnetic model of human falls. The model neglects articulated limb motion, torso–leg coordination, body rotation, stumbling recovery, self-occlusion, clothing-dependent scattering, and interactions with objects or the floor after contact. These factors can create additional micro-Doppler components that are not represented by a single rigid plate. In this study, such unmodeled variability is treated as a domain gap and is partially covered through randomization of geometry, initial velocity, effective-range normalization, amplitude, and noise. Thus, the model should be interpreted as a physics-informed first-order approximation for enacted trip falls, not as a general model of all fall morphologies.

The plate falls in the positive *z*-direction. Let the fall (tilt) angle be θ with 0≤θ≤π/2 and the fall azimuth be ϕ with −π≤ϕ<π. Consider a point on the plate at (x,ycosθ,ysinθ) and a Doppler sensor located at (−dsinϕ,h,−dcosϕ). The distance L(t) between them is

(8) $$\begin{array}{ccc} L(t) & = & \sqrt{{\left(x+d\sin\phi \right)}^{2}+{\left(y\cos\theta -h\right)}^{2}+{\left(y\sin\theta +d\cos\phi \right)}^{2}}\\ & = & \sqrt{X^{2}+Y^{2}+A^{2}} \end{array}$$

where, for readability, we define X=x+dsinϕ, Y=y+(dsinθcosϕ−hcosθ), and $A^{2}=h^{2}+d^{2}\cos^{2}\phi -{\left(d\sin\theta \cos\phi -h\cos\theta \right)}^{2}$. Integrating L(t) over the plate area gives the area-integrated geometric range

(9) $$\begin{array}{ccc} \tilde{D}\left(t\right) & & =\int_{Y_{1}}^{Y_{2}}\int_{X_{1}}^{X_{2}}\sqrt{X^{2}+Y^{2}+A^{2}}\;dx\;dy\\ & & =\frac{1}{18}[[-6A^{3}\tan^{-1}\;\left(\frac{xy}{A\sqrt{A^{2}+x^{2}+y^{2}}}\right)\\ & & +3x\left(3A^{2}+x^{2}\right)\log\;\left(\sqrt{A^{2}+x^{2}+y^{2}}+y\right)\\ & & +3y\left(3A^{2}+y^{2}\right)\log\;\left(\sqrt{A^{2}+x^{2}+y^{2}}+x\right)\\ & & -y\;\left(-6x\sqrt{A^{2}+x^{2}+y^{2}}+6A^{2}+y^{2}\right)\\ & & +6A^{3}\tan^{-1}\;\left(\frac{y}{A}\right){]}_{X_{1}}^{X_{2}}{]}_{Y_{1}}^{Y_{2}}. \end{array}$$

The effective range used in the Doppler phase is then defined as

(10) $$\begin{array}{c} D\left(t\right)=K\tilde{D}\left(t\right), \end{array}$$

with limits $X_{1}=-\frac{W}{2}+d\sin\phi$, $X_{2}=\frac{W}{2}+d\sin\phi$, $Y_{1}=\left(d\sin\theta \cos\phi -h\cos\theta \right)$, and $Y_{2}=H+\left(d\sin\theta \cos\phi -h\cos\theta \right)$. The closed form in (9) was obtained using Mathematica.

Because $\tilde{D}\left(t\right)$ has units of $m^{3}$, *K* has units of $m^{-2}$ and should be interpreted as an empirical effective-range normalization factor. For a uniformly scattering plate, K=1/(WH) corresponds to the area-averaged range. In practice, the dominant scattering center can deviate from the uniform area average because of body shape, posture, clothing, and aspect angle; therefore, *K* is randomized during synthesis to cover this residual geometric uncertainty.

### 3.2. Fall-Angle Dynamics

To use the model in Section 3.1, we express the fall angle θ as a function of time *t*. Let *I* be the moment of inertia and *T* the torque acting on the plate. The equation of motion is

(11) $$\begin{array}{c} I\frac{d^{2}\theta }{dt^{2}}=T. \end{array}$$

For a plate of mass *m*,

(12) $$\begin{array}{c} I=\frac{1}{3}mH^{2},\;T=\frac{1}{2}mgH\sin\theta , \end{array}$$

and substituting (12) into (11) gives

(13) $$\begin{array}{c} \frac{d^{2}\theta }{dt^{2}}=\frac{3g}{2H}\sin\theta . \end{array}$$

Because an enacted fall evolves from θ=0 to approximately π/2, the small-angle approximation sinθ≈θ is not valid over the full fall trajectory. Therefore, the simulator uses the nonlinear equation in (13) as the primary kinematic model and solves it numerically with the initial conditions

(14) $$\begin{array}{c} \theta \left(0\right)=0,\;\dot{\theta }\left(0\right)=\frac{v_{0}}{H}, \end{array}$$

until θ(t) reaches π/2. A fourth-order Runge–Kutta scheme with a 1-ms time step was used for synthesis, consistent with the 1-kHz sampling rate of the measured baseband data.

The linearized equation obtained by setting a=3g/(2H) and sinθ≈θ has the closed-form solution

(15) $$\begin{array}{c} \theta_{\mathrm{lin}}\left(t\right)=\frac{v_{0}}{H\sqrt{a}}\sinh\;\left(\sqrt{a}\;t\right). \end{array}$$

This analytical expression is useful for interpreting the early phase of the motion but is not used as the default simulator. The effect of using the linearized rather than nonlinear trajectory is quantified in Section 5.1.

## 4. Experimental Protocol

This section presents two experiments designed to evaluate the effectiveness of the proposed model and learning pipeline within a controlled setting. In Experiment I, we assess whether the proposed trip-fall model reproduces measured fall dynamics by comparing simulated and measured spectrograms under fixed sensor height, subject–sensor distance, and fall direction. The comparison is quantified using image-level and ridge-level similarity metrics. In Experiment II, we examine whether a model trained exclusively on simulated data can detect measured trip falls. To this end, we train on synthetic data generated from (i) the proposed trip-fall model, (ii) a kinematic walking model, and (iii) a respiration model, and then perform three-class discrimination among trip fall, walking, and seated quiet breathing.

This evaluation intentionally focuses on enacted trip falls because they can be performed safely and reproducibly under the approved protocol while still producing rapid forward loss-of-balance motion relevant to CW Doppler fall detection. The present protocol does not include slip falls, step-down falls, partial falls, assisted falls, or falls involving furniture. In addition, the non-fall classes are walking and seated quiet breathing; fall-like activities of daily living such as sitting down, bending, kneeling, intentionally lying down, and stumbling-recovery are not evaluated in the current dataset. The reported performance should therefore be interpreted as controlled feasibility evidence for the present three-class task, not as deployment-ready validation across all fall and non-fall activities.

All measurements were acquired using a commercial 24 GHz CW Doppler radar sensor (IPS-155, InnoSenT GmbH, Donnersdorf, Germany). Each sensor outputs 40 mW nominal power (100 mW max), has 20 dB gain, and a −3 dB full beamwidth of 70° (horizontal) ×36° (vertical). The in-phase (I) and quadrature (Q) baseband signals were filtered and amplified, then simultaneously digitized at 1 kHz using a USB A/D converter (AI-16068AY-USB, CONTEC, Tokyo, Japan) and logged to a PC. Falls are commonly categorized into three broad types—trip, slip, and step-down [35]. In this study, participants enacted trip falls; slip and step-down falls will be addressed in future work. All procedures involving human participants were approved by the Chuo University Research Ethics Committee (Approval No. 2024-063) and adhered to the relevant ethical guidelines. Five young adults participated in Experiment I (Young group; *N* = 5; 3 males, 2 females; age: 27.4 ± 7.6 years; height: 168.6 ± 13.7 cm; shoulder width: 40.8 ± 2.8 cm). Experiment II included these young participants and five older adults (Older group; *N* = 5; 3 males, 2 females; age: 64.6 ± 3.6 years; height: 164.6 ± 7.1 cm; shoulder width: 39.6 ± 2.9 cm). In total, ten participants (*N* = 10) were analyzed in Experiment II.

### 4.1. Experiment I

**Figure 2:**
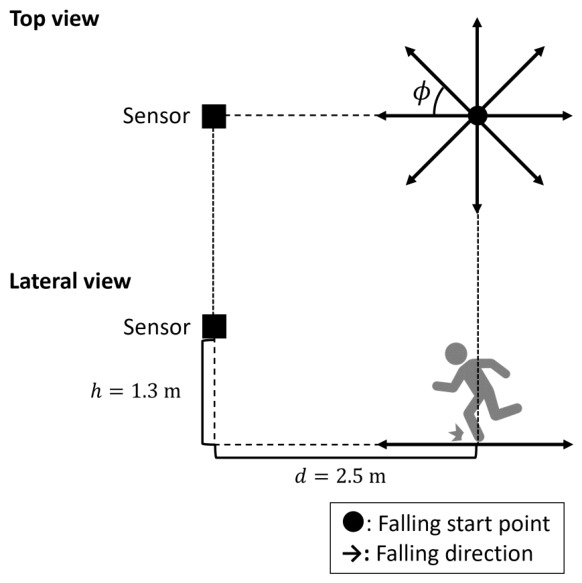
Geometry of the radar observation setup for Experiment I.

Experiment I evaluates the fidelity of the simulated Doppler spectrograms against real observations. As illustrated in Figure 2, each participant initiated the action from a point 2.5 m from the radar and fell toward direction ϕ. The radar was mounted at a height of 1.3 m. Because antipodal or symmetric orientations yield equivalent micro-Doppler patterns (e.g., $45^{{}^{\circ}}$/315° and 90°/270°), we tested five directions: 0°, 45°, 90°, 135°, and 180°. For safety, an air mattress was placed along the fall direction.

### 4.2. Experiment II

**Figure 3:**
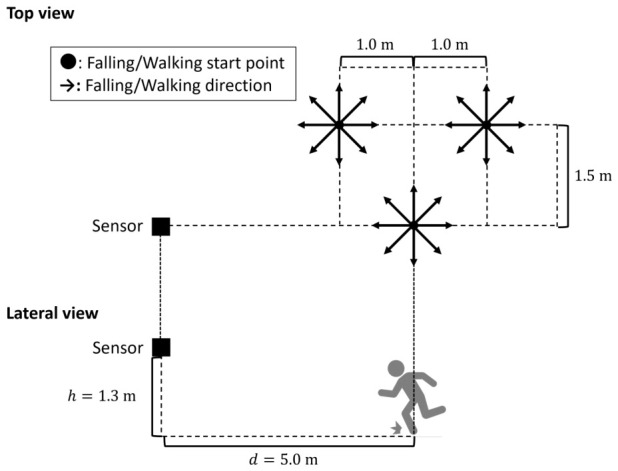
Geometry of the radar observation setup for Experiment II.

Experiment II examines the applicability of the proposed approach to fall detection. As illustrated in Figure 3, each participant performed fall, walking, and seated breathing actions two times for each direction ϕ at each of three distinct start locations (A, B, C) varying in both range and azimuth relative to the radar.

For fall, the participant enacted a trip fall toward direction ϕ starting from the initial posture. For walking, the participant took four steps along direction ϕ. For breathing, the participant sat facing direction ϕ and breathed quietly for 10 s. Because the apparent aspect varies with start location, each location was tested over eight directions: 0°, 45°, 90°, 135°, 180°, 225°, 270°, and 315°. For safety, an air mattress was placed along the fall direction as in Section 4.1.

In Experiment II, the experiment was conducted with a total of 10 participants, including the five participants in Experiment I. In our simulator, the parameter H corresponds to the subject’s body height, and W serves as a proxy for the subject’s lateral body extent (approximated here by shoulder width). Notably, the observed participant ranges are contained within the synthesis randomization ranges (H∈[150,190] cm and W∈[30,60] cm), supporting evaluation across a nontrivial spread of body sizes and ages.

#### Fall Detection

For fall detection, we adopt a baseline approach that couples short-time Fourier transform (STFT) spectrograms with a ResNet-18 classifier [36]. The model was trained using the Adam optimizer with a learning rate of 0.001 and a batch size of 32. All inputs were resized to 224 × 224 pixels. Training was conducted for 100 epochs with a weight decay of 1 × 10^−4^. All experiments were implemented in PyTorch 2.11 and run on an NVIDIA RTX 4090 GPU. This workstation setup was used to ensure reproducible model training and analysis; it should not be interpreted as evidence of embedded or edge-device deployment readiness. Our contribution focuses on demonstrating the effectiveness of simulation-only training under a fixed and common learning pipeline; identifying the optimal lightweight architecture for deployment is left to future work.

To clarify the fairness of the comparison between simulation-only training and real-data training, we matched the learning pipeline as closely as possible across conditions. Specifically, the same STFT-based input representation, frequency range (0–500 Hz), log-magnitude + min–max normalization, image resizing (224 × 224), ResNet-18 architecture, optimizer (Adam), learning rate, batch size, number of epochs, and weight decay were used in both conditions. In addition, evaluation was conducted on the same measured test framework under the corresponding LNSO/LNLO protocols. Thus, the principal factor changed between the two settings was the source of the training data (synthetic or measured).

We note, however, that the amount of available training data was not matched exactly between conditions. The simulation-only setting can generate substantially more diverse samples through parameter randomization, whereas the real-data setting is inherently constrained by the number of participants, locations, and repetitions. Therefore, the present comparison should be interpreted as an assessment of practical training paradigms under realistic data-availability constraints, rather than as a strictly size-matched comparison.

Dataset size and preprocessing: In Experiment II, each participant contributed 48 trials per class (2 repetitions × 8 directions × 3 start locations), yielding 480 fall trials, 480 walking trials, and 480 breathing trials across all 10 participants (1440 trials total). Each trial was segmented to cover the action interval (from the instructed start to the end of the motion), and converted to a time–frequency representation using STFT with a 0.5 s window, 0.1 s hop, and a Hann window. We computed the STFT on the complex baseband signal s(t)=I(t)+jQ(t), retained the 0–500 Hz band, and used the magnitude spectrogram as the classifier input. Each spectrogram was normalized using log-magnitude + min-max and resized to 224 × 224 pixels for CNN-based models. The same preprocessing pipeline was applied to both simulated and measured baseband signals to ensure a consistent representation.

Training data for simulation-only learning were generated from three models. For fall, we used the proposed physics-based model. For walking, we extended a kinematic gait model [37] to radar simulation following the formulation in [38]. For respiration, we used a mathematical model of chest wall motion [39]. For each model, we synthesized large corpora across start locations and directions to test whether real falls can be detected without any real training examples.

During synthesis we randomized the following parameters. For the fall model: height H∈[150,190] cm (0.1 cm steps), width W∈[30,60] cm (0.1 cm steps), initial tip-velocity parameter $v_{0}\in \left[0.5,5.0\right]$ (0.01 steps), and the effective-range normalization parameter $K\in \left[0.01,5\right]\;m^{-2}$ (0.01 steps). For the gait model [38], we varied height and width over the same ranges and swept the walking-speed parameter RV∈[0.5,3.0] (0.01 steps) in accordance with [37]. For the respiration model [39], we varied the breathing frequency over 0.1–0.5 Hz (0.01 Hz steps). We added white noise to the synthetic waveforms as a form of data augmentation, following denoising/robustness practices in the radar HAR literature to better match measurement conditions [40].

For comparison, we also trained on real data using a leave-N-subjects-out (LNSO) protocol, where N subjects were held out for testing, and the remaining 10-N subjects were used for training (N = 1, …, 9), and evaluated fall detection accuracy to benchmark the proposed approach. In addition, we conducted leave-N-locations-out (LNLO) evaluations in which one or two locations were withheld during training and models were tested on the unseen location(s) to assess sensitivity to distribution shift induced by motion start location and direction.

Baseline classifiers: To examine whether the benefit of simulation-only training generalizes across model families, we also trained additional classifiers following representative prior approaches (CNN [41], CNN [42], and an SVM [43]) and evaluated them under the same preprocessing and LNSO/LNLO protocols. These baselines based on the CNN use the identical spectrogram representation, and those based on SVM use the feature values based on the observed signals as input; model-specific details follow the corresponding references to ensure faithful comparison.

Statistical analysis and additional computational baselines: For the ResNet-18 comparison, 95% confidence intervals were estimated using participant-clustered bootstrap resampling with 10,000 resamples. Because multiple trials from the same participant are not independent, participants rather than individual trials were treated as the resampling units. The difference between simulation-only training and real-data training was evaluated using a paired subject-level permutation test based on held-out-subject macro-F1 scores. To address the effect of training-data size and augmentation without collecting additional human-subject data, we also evaluated real-data training with spectrogram augmentation, size-matched simulation training, and simulation pretraining followed by real-data fine-tuning. The augmentation operations were amplitude scaling, time shift, additive measured-noise mixing, and random time–frequency masking.

## 5. Results

### 5.1. Results of Experiment I

**Figure 4:**
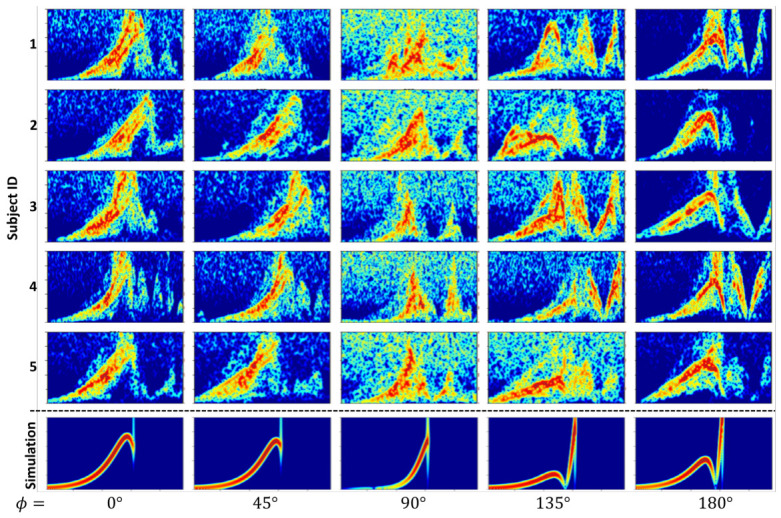
Measured vs. simulated Doppler spectrograms of trip falls across five fall directions. The color intensity in the spectrograms represents the normalized spectral magnitude. The horizontal dotted line separates the measured spectrograms (top five rows) from the simulated spectrograms (bottom row).

Figure 4 shows the measured spectrograms alongside the corresponding simulated spectrograms for five participants and multiple fall directions. The vertical axis shows frequency (0–500 Hz); the horizontal axis shows time; color encodes spectral magnitude. STFT used a 0.5 s window, 0.1 s hop, and a Hann window. The representative simulation shown uses H=150 cm, W=50 cm, range d=2.5 m, sensor height h=1.3 m, $v_{0}=2.0$, and the nonlinear fall-angle model in Section 3.2. Since the duration of the fall differs by participant, measured and simulated spectrograms were time-normalized before the quantitative comparison.

Overall, the simulated spectrograms capture the dominant time–frequency characteristics of measured trip falls. For instance, at ϕ=90° the measured spectrograms display stronger noise components; this is consistent with reduced radial velocity relative to the radar line of sight, which lowers SNR and makes non-target components more prominent. In some trials, additional frequency components appear after the fall in measured data but not in simulation; inspection revealed that these arose from the rebound of the safety air mattress, which introduces transient artifacts.

To quantify this visual observation, we computed four similarity metrics between time-normalized measured and simulated spectrograms: structural similarity index (SSIM), Doppler-ridge mean absolute error (MAE), peak-Doppler error, and spectral-centroid error. The Doppler ridge was extracted as the frequency bin with maximum log-magnitude at each time frame after suppressing the static/near-zero-frequency component (Table 1 and Table 2).

These quantitative results support the visual observation that the simulator reproduces the dominant Doppler-ridge structure of enacted trip falls. At the same time, the larger errors at 90° indicate that the model remains less accurate when radial velocity is small and measured signals are dominated by noise, clutter, or post-contact artifacts. Thus, Experiment I validates the simulator as a first-order physics-informed approximation, not as a complete model of noise, multipath, clutter, hardware imperfections, or human biomechanics.

### 5.2. Results of Experiment II

Table 3 compares the macro-F1 scores of the ResNet-18 [36] model used in this study with those of other CNN-based [41,42] and SVM-based [43] approaches. The comparison was conducted under four training conditions: (i) training using only simulated data (proposed method); (ii) training using real data (Realdata); (iii) training using real data with one location out (Realdata, LNLO = 1); and (iv) training using real data with two locations out (Realdata, LNLO = 2). Confidence intervals were estimated using participant-clustered bootstrap resampling.

The ResNet-18-based model achieved the highest performance. In addition, for all tested classifiers, training on simulated data yielded higher performance than training on the available real data within the present dataset and evaluation framework. Because the preprocessing pipeline, network architecture, and optimization settings were fixed across conditions, Table 3 mainly reflects differences in training-data source and diversity within the present dataset. At the same time, the synthetic-data condition benefits from scalable sample generation through domain randomization [44]. Accordingly, these results should be interpreted as evidence of a practical advantage under limited real-fall data availability, rather than as proof that simulation-only training is universally superior under perfectly size-matched conditions.

**Figure 5:**
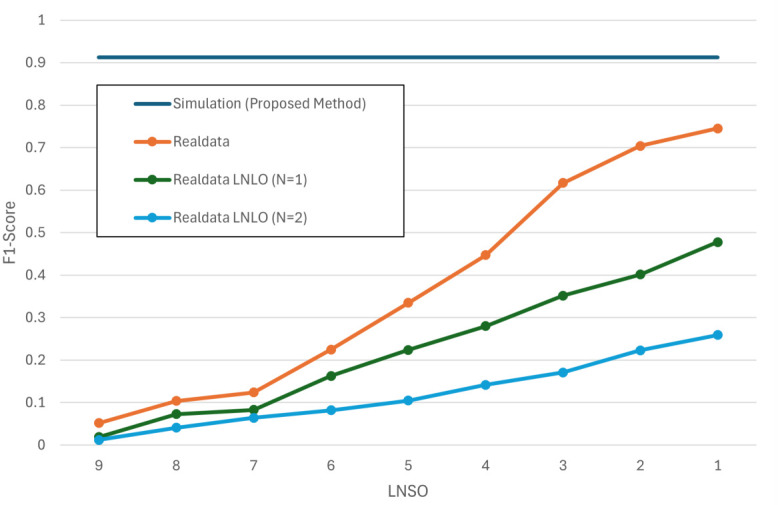
Macro-F1 versus *N* (the number of held-out subjects) under leave-*N*-subjects-out (LNSO) and leave-*N*-locations-out (LNLO) for four training regimes.

Next, we examine the effect of the number of subjects used for training on real data using the ResNet-18 model, which demonstrated the best performance. Figure 5 summarizes fall-recognition performance for four settings: (i) the proposed simulation-only training (Simulation); (ii) real-data training (Realdata); (iii) real-data training with one location withheld (Realdata *w*/*o* one area); and (iv) real-data training with two locations withheld (Realdata *w*/*o* two area). The vertical axis shows the macro-F1 score; the horizontal axis shows *N*, the number of held-out subjects in LNSO. The simulation-only result is a single reference value independent of LNSO because the model is trained solely on synthetic data and tested on all measured participants.

Table 4 lists the detailed macro scores of the simulation-only and real-data training results for the LNSO N=1 setting. The simulation-only model achieved a macro-F1 of 0.912 [95% CI: 0.883–0.936], whereas the real-data LNSO baseline achieved 0.748 [95% CI: 0.691–0.805]. A paired subject-level permutation test indicated a statistically significant difference between these two conditions (p=0.006). This result should be interpreted as evidence that the proposed simulation pipeline is useful when measured fall data are scarce, not as a claim that synthetic data are always superior to real data.

To further clarify whether the advantage comes only from synthetic sample size, Table 5 compares additional computational baselines generated from the existing dataset and simulator. Real-data augmentation improved the real-data baseline but did not close the gap to the full simulation-only model. Size-matched simulation training, in which the number of synthetic samples was matched to the available real training samples, also improved over real-only training. Simulation pretraining followed by real-data fine-tuning produced the highest value in this auxiliary analysis, suggesting that the proposed simulator may be useful not only for simulation-only training but also for few-shot adaptation.

**Figure 6:**
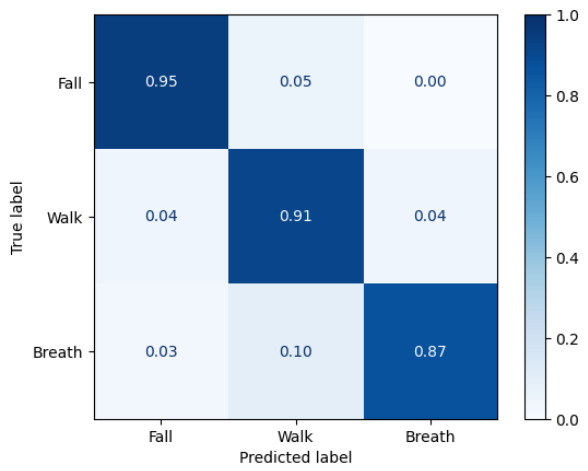
Normalized confusion matrix of the simulation-only training result.

**Figure 7:**
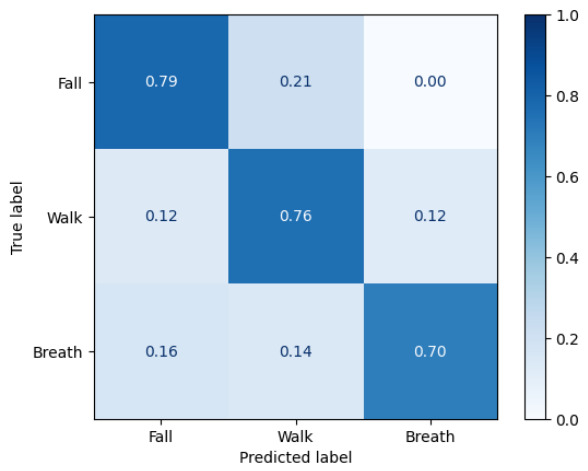
Normalized confusion matrix of the real-data training result.

Figure 6 and Figure 7 show the normalized confusion matrices of the simulation-only and real-data training results, respectively. To isolate the contribution of the proposed trip-fall model, Table 6 reports fall-vs-nonfall metrics and ablation results. The full simulator achieved a fall F1 of 0.940 and a false-alarm rate of 0.035 in this analysis. Removing domain randomization or fall-direction randomization reduced fall recall and increased false alarms, supporting the role of the proposed fall-model randomization in simulation-to-real transfer.

Table 3 and Figure 5 also illustrate that, under LNLO, macro-F1 deteriorates sharply when withholding one or two start locations, indicating strong dependence on sensor–subject geometry and start location in radar-based recognition. These findings suggest that achieving high-accuracy recognition across positions and directions using only real data may require substantially larger datasets spanning more diverse fall patterns, directions, velocities, and locations. The present results therefore support the usefulness of simulation-driven learning under controlled but geometrically varied conditions.

## 6. Discussion

A model trained solely on simulated spectrograms—generated by coupling a physics-informed trip-fall model with a CW radar observation model—achieved a macro-F1 of 0.912 on the measured dataset collected from ten participants across three start locations and eight fall directions. In contrast, the best real-data LNSO baseline achieved 0.748, and performance decreased markedly under LNLO when one or two start locations were withheld. These trends are consistent with the well-known geometry dependence of radar micro-/range-Doppler representations, including sensitivity to aspect angle and installation [15,16,17].

A point that warrants careful interpretation is the fairness of the comparison between simulation-only and real-data training. In this study, the model architecture, preprocessing pipeline, optimization settings, and measured test framework were controlled across conditions. However, the number and diversity of available training samples were not matched exactly because the synthetic pipeline can generate parameter-varied data at low cost, whereas the measured dataset is limited by practical and ethical constraints. Accordingly, the results should be interpreted as evidence of the practical advantage of simulation-driven learning under limited real-fall data availability, rather than as proof that synthetic data are universally superior under perfectly size-matched conditions. The auxiliary size-matched and augmented baselines in Table 5 partially address this issue, but broader comparisons with GAN-based synthesis, explicit domain adaptation, and transfer-learning methods remain future work.

The simplified fall model also requires careful interpretation. The single-plate representation captures the gross radial-velocity evolution of rapid trip-fall descent, which is a dominant component of the CW Doppler spectrogram. It does not model articulated limb motion, torso–leg coordination, body rotation, stumbling recovery, partial falls, assisted falls, furniture interaction, self-occlusion, or floor-contact dynamics. The sensitivity analysis in Table 6 suggests that randomization of the fall-model parameters is important for transfer, but it does not eliminate the need for richer human and scattering models. Future work should incorporate articulated human motion, motion-capture-driven validation, and more realistic propagation and hardware effects.

The current evaluation is also limited in activity scope. Walking and seated quiet breathing are spectrally distinct from falls, so the present three-class problem is easier than deployment-oriented fall detection against fall-like activities of daily living. The reported performance should therefore not be interpreted as evidence of robustness to sitting down, bending to pick up an object, kneeling, intentionally lying down, stumbling and recovering, or other rapid non-fall postural transitions. These activities are essential next-step negative classes for estimating false-alarm rates in practical systems.

A residual failure mode arises when falls occur nearly orthogonal to the radar line-of-sight. As shown in Section 5.1, measured falls at such orientations can produce small radial velocities and reduced SNR, whereas the simulator remains idealized. Environmental clutter, moving fans, pets, caregivers, multipath, and multi-person overlap can further perturb CW Doppler signals. Since a single CW radar does not provide range or angle separation, deployment in cluttered or multi-person environments would likely require multi-view sensing, additional modalities, or stronger signal separation methods.

### 6.1. Computational Scope and Deployment Limitations

The online pipeline was profiled only on a workstation equipped with an AMD Ryzen Threadripper PRO 5995WX CPU and an NVIDIA RTX 4090 GPU. With a 0.5 s STFT window and a 0.1 s hop, the system outputs decisions at 10 Hz, with an inherent buffering latency of at least 0.5 s. The per-update compute time on this workstation was 0.9 ± 0.2 ms for the STFT/spectrogram update and 1.8 ± 0.3 ms for ResNet-18 inference, resulting in 2.7 ± 0.4 ms end-to-end runtime. These values demonstrate algorithmic real-time feasibility on a high-performance workstation only. We did not evaluate memory footprint, quantization, CPU-only latency, low-power embedded processors, or edge accelerators in this study. Therefore, no claim is made that the current ResNet-18 implementation is ready for embedded deployment in homes or elderly-care facilities. Lightweight architectures, compression, quantization, and edge-hardware benchmarking are important future work.

### 6.2. Relation to Wi-Fi CSI-Based Privacy-Preserving Sensing

Wi-Fi CSI-based sensing is another device-free and privacy-preserving approach to indoor human activity recognition. Recent studies have demonstrated low-cost indoor activity recognition using Wi-Fi channel state information and real-time deep-learning frameworks for smart environments [45,46]. Compared with Wi-Fi CSI, CW Doppler radar provides a more direct measurement of radial velocity and micro-Doppler signatures, which is advantageous for interpreting rapid body motion. However, CW Doppler radar requires dedicated sensor placement and is sensitive to aspect angle; a single CW sensor also lacks range/angle separation. Wi-Fi CSI can potentially reuse existing wireless infrastructure and reduce hardware cost, but its performance depends on access-point/receiver geometry, CSI extraction capability, driver/device support, and environmental multipath. Thus, radar and Wi-Fi CSI involve different trade-offs rather than a simple superiority relationship.

Although the proposed approach showed strong simulation-to-real transfer in the present experiments, the findings should be interpreted within the scope of the evaluated participants, fall type, activities, environment, radar device, and measurement configurations. The study demonstrates the controlled feasibility of simulation-only training for enacted trip-fall detection, not general deployment readiness. Future work will (i) incorporate multi-segment human and scattering models; (ii) evaluate fall-like activities of daily living; (iii) test cross-environment, cross-device, and multi-person scenarios; (iv) compare with explicit domain adaptation and generative sim-to-real methods; and (v) investigate lightweight edge implementations.

## 7. Conclusions

We presented a controlled feasibility study of simulation-only training for CW Doppler radar-based trip-fall detection. The proposed framework couples a physics-informed kinematic trip-fall model with a CW Doppler observation model and trains detection models exclusively on simulated spectrograms. In Experiment I, quantitative similarity metrics showed that the simulator reproduced the dominant Doppler-ridge structure of measured enacted trip falls, although noise, clutter, multipath, hardware imperfections, and post-contact artifacts were not fully modeled. In Experiment II, the proposed approach achieved a macro-F1 score of 0.912 [95% CI: 0.883–0.936] on measured data collected from ten participants across three start locations and eight directions, exceeding the available real-data-trained baseline within the same evaluation framework.

These findings suggest that physics-informed simulation with domain randomization can reduce dependence on measured trip-fall samples under limited-data conditions. However, the results are limited to enacted trip falls and controlled three-class discrimination among trip fall, walking, and seated quiet breathing. They do not establish robustness to slip falls, step-down falls, partial or assisted falls, fall-like activities of daily living, multi-person scenarios, environmental clutter, different radar devices, or embedded deployment. Future work will integrate articulated human and scattering models, evaluate fall-like daily activities, examine cross-environment and cross-device generalization, compare with explicit sim-to-real/domain-adaptation methods, and combine simulation pretraining with lightweight real-data adaptation for practical deployment.

## Figures and Tables

**Table 1:** Quantitative similarity between measured and simulated trip-fall spectrograms in Experiment I. Values are mean ± SD across participants.

Fall Direction	SSIM	Doppler-Ridge MAE (Hz)	Peak-Doppler Error (Hz)	Spectral-Centroid Error (Hz)
0°	0.812±0.041	18.7±5.6	26.4±8.9	13.1±4.2
45°	0.791±0.052	22.3±7.1	29.7±10.4	15.4±5.0
90°	0.692±0.087	34.5±11.8	43.8±16.6	22.9±7.8
135°	0.774±0.063	25.9±9.2	34.2±13.1	17.8±6.1
180°	0.837±0.038	17.6±4.9	24.5±7.7	12.6±3.9
Mean	0.782±0.076	24.6±8.7	31.6±12.4	16.4±6.3

**Table 2:** Effect of fall-angle dynamics on simulation-to-real similarity and classification performance.

Kinematic Model	Duration Error (%)	Peak-Doppler Error (Hz)	Ridge MAE (Hz)	SSIM	Macro-F1
Linearized sinθ≈θ	11.8±4.1	41.2±18.4	31.6±10.8	0.741±0.087	0.903
Nonlinear numerical integration	6.3±2.7	30.5±15.0	24.1±8.2	0.782±0.076	0.912

**Table 3:** Macro-F1 comparison with other approaches. Values in brackets denote 95% confidence intervals.

Model	Simulation (Proposed)	Realdata	Realdata (LNLO = 1)	Realdata (LNLO = 2)
ResNet-18 [36]	0.912 [0.883, 0.936]	0.748 [0.691, 0.805]	0.477 [0.412, 0.541]	0.259 [0.211, 0.316]
CNN [41]	0.878 [0.842, 0.910]	0.704 [0.645, 0.761]	0.356 [0.299, 0.419]	0.233 [0.181, 0.292]
CNN [42]	0.865 [0.829, 0.899]	0.734 [0.681, 0.790]	0.412 [0.351, 0.471]	0.247 [0.198, 0.304]
SVM [43]	0.593 [0.541, 0.646]	0.368 [0.310, 0.424]	0.233 [0.187, 0.289]	0.156 [0.119, 0.204]

**Table 4:** Performance metrics for simulation-only training and real-data training (LNSO, N=1). Confidence intervals were estimated by participant-clustered bootstrap resampling.

Training Data	Macro-F1	95% CI	Accuracy	Precision	Recall
Simulation (Proposed)	0.912	[0.883, 0.936]	0.911	0.914	0.911
Realdata	0.748	[0.691, 0.805]	0.746	0.756	0.746

**Table 5:** Additional training-regime baselines using the existing measured data and simulator.

Training Regime	Training Size	Augmentation	Macro-F1 [95% CI]
Real only	1296 trials/fold	none	0.748 [0.691, 0.805]
Real + augmentation	1296 trials/fold + augmentation	yes	0.781 [0.724, 0.831]
Simulation, size-matched	1296 synthetic samples	yes	0.861 [0.820, 0.899]
Simulation, full	14,400 synthetic samples	yes	0.912 [0.883, 0.936]
Simulation pretraining + real fine-tuning	mixed	yes	0.928 [0.902, 0.951]

**Table 6:** Fall-specific metrics and ablation analysis. False-alarm rate denotes non-fall trials classified as fall.

Training/Synthesis Condition	Macro-F1	Fall Precision	Fall Recall	Fall F1	False-Alarm Rate
Full proposed simulator	0.912	0.931	0.950	0.940	0.035
Fixed fall parameters, no domain randomization	0.781	0.806	0.842	0.823	0.104
No fall-direction randomization	0.844	0.861	0.887	0.874	0.073
Linearized fall-angle model	0.903	0.921	0.944	0.932	0.041
Real-data LNSO baseline	0.748	0.738	0.790	0.763	0.140

## Data Availability

The data presented in this study are available on request from the corresponding author.

## Abbreviations

The following abbreviations are used in this manuscript:

CW	Continuous wave
FMCW	Frequency-modulated continuous wave
IR-UWB	Impulse Radio Ultra Wideband
LNSO	Leave-N-Subjects-Out
LNLO	Leave-N-Locations-Out
STFT	Short-Time Fourier Transform
CNN	Convolutional Neural Network
SVM	Support Vector Machine
